# Humanitarian health education and training state-of-the-art: a scoping review

**DOI:** 10.3389/fpubh.2024.1343867

**Published:** 2024-07-29

**Authors:** Awsan Bahattab, Monica Trentin, Ives Hubloue, Francesco Della Corte, Luca Ragazzoni

**Affiliations:** ^1^CRIMEDIM – Center for Research and Training in Disaster Medicine, Humanitarian Aid, and Global Health, Università del Piemonte Orientale, Novara, Italy; ^2^Department for Sustainable Development and Ecological Transition, Università del Piemonte Orientale, Vercelli, Italy; ^3^Department of Translational Medicine, Università del Piemonte Orientale, Novara, Italy; ^4^Research Group on Emergency and Disaster Medicine, Vrije Universiteit Brussel, Brussels, Belgium

**Keywords:** capacity building, competencies, curriculum, humanitarian health, professionalization, relief work, training and education, simulation

## Abstract

**Introduction:**

In the past decade, humanitarian emergencies have been increasing, leading to an higher demand for humanitarian health professionalization. Education and training are critical for preparing these workers to provide effective care during crises. Understanding the current state-of-the-art in humanitarian health education is essential to inform research and development of future educational programs. This review surveys the peer-reviewed literature to provide insights into the current thinking in the field.

**Methods:**

A review was conducted in March 2023 and updated in May 2024 using PubMed, Web of Science, Scopus, and Education Resources Information Center databases for English-language peer-reviewed articles published since January 2013. The review followed the Joanna Briggs Institute methodology for scoping reviews and adhered to the Preferred Reporting Items for Systematic Reviews and Meta-Analyses extension for Scoping Reviews (PRISMA-ScR). Data were analyzed using qualitative content analysis and presented as a narrative descriptive summary.

**Results:**

After screening, 32 articles met the inclusion criteria. The themes of the selected articles focus on education and training frameworks, mapping, and programs. Despite the growing opportunities, most education and training programs are based in the Global North. The gaps identified include a lack of standardized curriculum or competency frameworks and evaluation frameworks to guide the development and evaluation of further standardized training programs. Interdisciplinary and collaborative partnerships, iterative design, and mixed teaching methods and modalities, including e-learning, facilitated successful training. However, logistical and technical constraints and the lack of standardized training frameworks were barriers to developing, implementing, and evaluating such training programs.

**Conclusion:**

This review provides an overview of the humanitarian health education trends over the last decade and identifies key areas for future educational development and research. The findings emphasize the importance of adapting interdisciplinary and collaborative partnerships and prioritizing the training of local staff through regional centers, local leadership, and accessible e-learning, including e-simulation. The review also highlights the need for continued research and evaluation of humanitarian health education and training programs with standardized metrics to evaluate training programs and identify areas for improvement. These steps will help ensure that humanitarian health professionals receive adequate training to provide effective healthcare in crisis situations.

## 1 Introduction

The upward trend of humanitarian emergencies has doubled the number of people affected by humanitarian crises in the last 4 years (1). Most of these crises are complex (1) and political in nature and require an international response due to a total or considerable breakdown of authority (2). Compared to the past, such crises have become more frequent, severe (3, 4) and protracted (1), lasting an average of 7 years (5) and resulting in serious public health negative consequences (6–8). Today, one in every 23 people needs humanitarian assistance due to conflict, climate crisis, and health epidemics such as COVID-19 and cholera (1). As a result, the humanitarian field has expanded in the last decade, with an increase in the number of humanitarian organizations and workers – mainly at the national level – by 10 and 40%, respectively (9). This expansion of the humanitarian field was coupled with increased demands for the professionalization of humanitarian assistance (10) and evidence-based public health interventions (11). Such a request was also raised directly by humanitarian health professionals (12), especially after the criticism they had received due to the consistent gaps in humanitarian response (13–15).

Health professionals play a critical role in responding to humanitarian emergencies by preventing excess deaths and addressing the “secondary toll” on public health, which often surpasses direct causalities (6–8, 16, 17). Hence, to provide an effective and quality humanitarian response, they need to be equipped with the essential skills and knowledge (14). Given that humanitarian health is constantly evolving, with new challenges and emerging best practices, there is an even greater need for appropriate education and training that embraces these challenges.

Although the exact number of humanitarian health workers and their specific competencies are unknown, the health sector is by far the first for job vacancies and the second for aid recipients (9). Like other health careers, education and training are essential elements of humanitarian health professionalization (18).

Historically, the training courses in humanitarian health were provided primarily by the International Committee of the Red Cross (ICRC) since the 1970s, followed by Médecins Sans Frontières (MSF). It was not until the late 1990s that academic institutions, influenced by disaster medicine, began to provide humanitarian health courses (19) to address the new challenges of sudden onset disasters, public health emergencies of international concern, and complex humanitarian emergencies (20). Although the response to disasters and complex humanitarian emergencies are somewhat interlinked, their operational and legal aspects are distinct. Hence, training needs are also distinct (20, 21), which left the existing training for disaster medicine falling short of humanitarian context reality (20). Moreover, effective humanitarian health response necessitates collaboration across various sectors, including but not limited to Health, Water, Sanitation, and Hygiene (WaSH), agriculture, Law, to address these complex challenges comprehensively (12). Therefore, integrating interprofessional and interdisciplinary collaboration into educational and training programs is crucial to ensure comprehensive preparedness and response in humanitarian contexts.

Therefore, this review is intended to provide timely overview on the education and training literature of international humanitarian public health response to inform future educational research and stimulate the development of future educational programs, which ultimately will ensure that health workers are adequately prepared to respond to the evolving challenges of humanitarian emergencies.

## 2 Methods

### 2.1 Design

This scoping review method and reporting were based on the Johanna Briggs Institute methodology for scoping reviews (22, 23) and the Preferred Reporting Items for Systematic Reviews and Meta-Analyses Extension for Scoping Reviews (PRISMA-ScR) checklist (24). Unlike systematic reviews, which address a relatively narrow range of quality-assessed studies, systematic scoping reviews enable dealing with broader questions, mapping the key concepts underpinning a research area and the main sources and types of evidence with a range of methodologies, and do not require quality assessment.

### 2.2 Search strategy

The literature search was conducted on March 2023, and updated on May 2024 on PubMed, WoS, Scopus, and ERIC databases. The search strategy began with a preliminary limited search of MEDLINE to identify relevant terms and keywords. The search included terms and keywords related to the two main concepts of this study: humanitarian health and education, combined using Boolean operators (AND/OR). Specifically, the keywords and terms included:

- Humanitarian Health: Terms such as “humanitarian public health,” “international humanitarian response,” “conflict-affected areas,” and “relief work.”
- Education/Training: Terms related to “competence,” “curricula,” “education,” “medical instruction,” “internships,” “residency,” “preparedness,” “teaching,” and “training.”

While the Boolean operator OR was used to include all variations within each concept (e.g., “Humanitarian Health” AND Education/Training), the operator AND was used to combine the two main concepts, ensuring that the search results included articles addressing both humanitarian health and education/training. The search strategy was tailored for each database to account for variations in indexing and search functionalities (Supplementary material 1).

The search results were imported into Rayyan Systematic Review Literature tool (25), and duplicates were removed. The initial screening of titles and abstracts was conducted independently by two reviewers based on the inclusion criteria. After this, the reviewers examined the full-text and applied the inclusion criteria. Any conflicting decisions during this phase were discussed and resolved in meetings between the co-investigators.

### 2.3 Eligibility criteria

This review was guided by the Joanna Briggs Institute “Population, Concept, Context” framework (22) (Table 1). To be eligible for inclusion, articles had to deal with education and training for international humanitarian public health response. In this review, “humanitarian” refers to low- and middle-income countries where a singular event or series of events, such as an armed conflict, disasters caused by natural or anthropogenic hazards, epidemic, or famine – have threatened the health, safety, or well-being of a large group of people and international humanitarian assistance is needed to support the affected population (26). Hence, any study that does not focus on training and education or is beyond the scope of the international humanitarian public health response has been excluded. This includes capacity-building activities, such as formal medical education programs in conflict-affected settings, or training focuses on single infectious disease.

**Table 1:** Inclusion and exclusion criteria.

	Inclusion criteria	Exclusion criteria
Population	The target group of this scoping review includes all health professionals who participate in international humanitarian missions to provide public health activities, including local and international respondents.	In-service staff training as part of a specific program or project implementation.
Context	International humanitarian health response.	Disaster medicine and management. Clinical medical and surgical skills. Undergraduate or postgraduate national medical education institutes.
Concept	Training and education programs of international humanitarian health respondents, including curriculum, competencies, guidelines, teaching, assessment, and evaluation methods and tools.	Training program whose specific aim was not the field of humanitarian health. Capacity-building activities other than education and training programs. Training programs focused on building capacity for a single-disease control and management.
Study	Peer-reviewed literature including experimental and quasi-experimental study designs, reviews descriptive and analytical observational study designs, and qualitative studies; and opinion papers.	Gray literature, newspapers, websites, brochures.
Language and full text	English language. Full-text available.	Non-English language. Full-text not available.

Since a preliminary review of gray literature revealed that the scope of humanitarian training and education information varies widely between different organizations, using different terminologies, and does not clearly focus on health—with most information about humanitarian health programs being web-based (news, courses repositories, databases) rather than gray-literature documents—our review includes only peer-reviewed literature. Additionally, most non-peer reviewed document about organization-specific competencies. While some are more generic for humanitarian field, other specific for health or other special topic related to health. However, it is not clear how these competencies are being used to inform the training programs development. Previous studies have shown that even competency-based programs used discipline-specific competencies instead of humanitarian competencies (12), and still, it is not clear if the humanitarian competencies are being used to inform humanitarian health training programs (27). A study that used humanitarian competencies framework for evaluation found misalignment between assessed competencies and actual fieldwork suitability of these competencies, when assessed by global rating. This indicates the need for competency framework to better reflect the realities of humanitarian work, particularly in terms of cultural and contextual adaptability (28). Finally, previous studies highlighted that the lack of standardized terminology and understanding of competency-based education frameworks, which create vagueness and inconsistent terminologies when they are used (29). To provide an overview of the current state of knowledge, we decided to include studies published from 2013 onwards.

### 2.4 Data extraction

Due to the varied focus and objectives of the publications related to humanitarian health education, we have considered an iterative process for data extraction.

First, an Excel sheet was developed to extract general characteristics from the included articles, to include information about the authors, publication year, study type, study objectives, and main findings of the studies. Furthermore, information about the characteristics of the training programs has also been extracted using specific sheets. Information about the course and simulation included training provider, location of the training, duration, topic, target audience, teaching delivery modality, teaching strategies and methods, students’ assessment, training evaluation, programs successful characteristics, and challenging for implementation. For simulations, information on the type of simulation, pre-simulation training, simulation scenario and tasks assigned during simulation was also extracted.

### 2.5 Analysis and reporting

To achieve the objective of this study, a qualitative content analysis was conducted (23). Initially, two investigators examined the objectives of all included articles. Based on these objectives, the articles were clustered into five categories: training needs and challenges, training opportunities mapping, curriculum, competency framework and skills, training programs.

The results of each category are presented as a narrative descriptive summary accompanied by tabulated and/or charted results, as appropriate.

### 2.6 Ethical considerations

No ethical board approval was necessary to conduct this literature review.

## 3 Results

**Figure 1:**
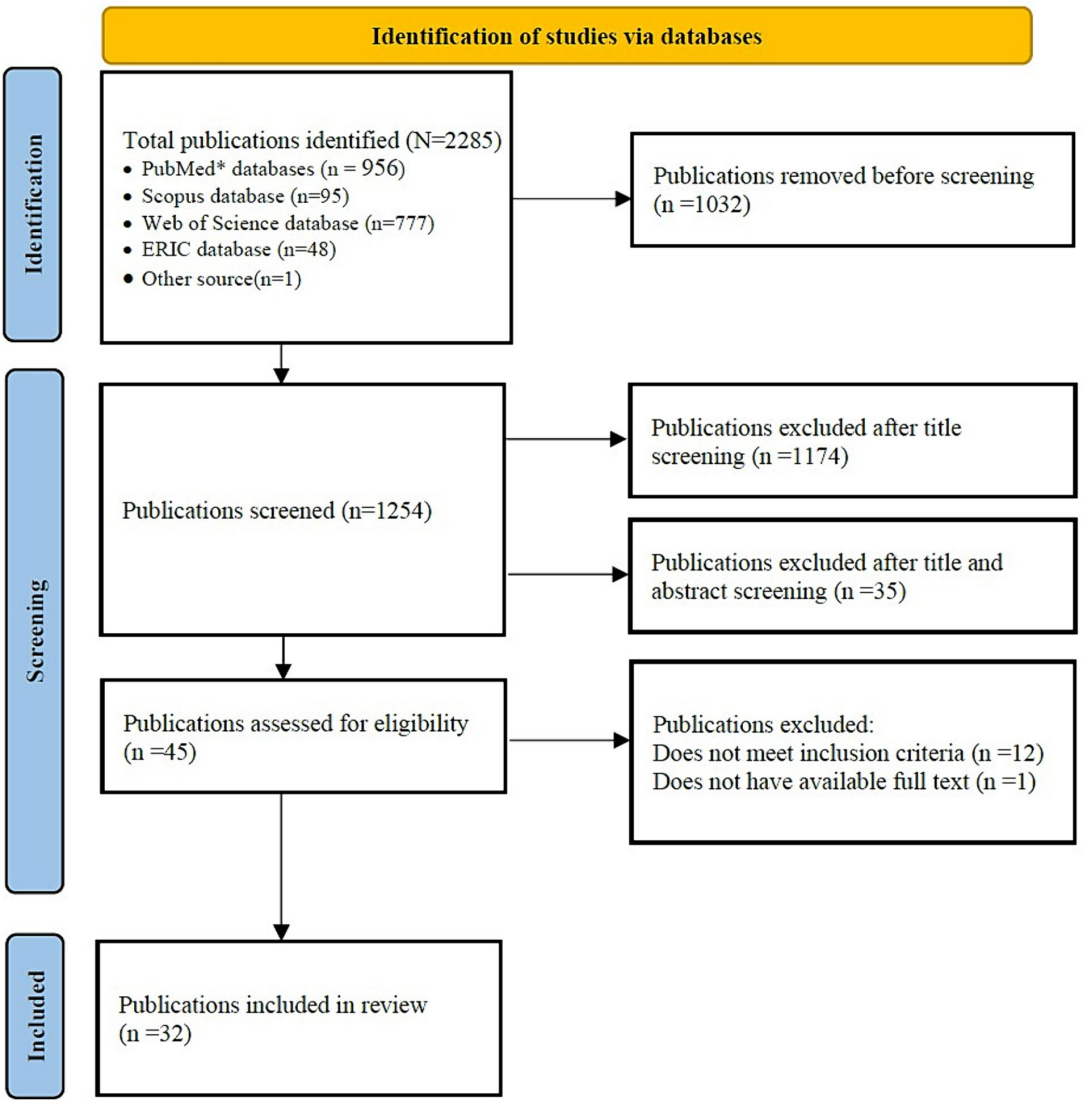
Flow chart diagram of the study selection process based on PRISMA [adapted from Page et al. (30)].

In total, database searches retrieved 2,285 articles. After the screening was completed, 32 articles were identified as meeting the inclusion criteria. Detailed information regarding the selection of sources of evidence can be found in the PRISMA diagram (Figure 1).

### 3.1 Characteristics of the publications

**Figure 2:**
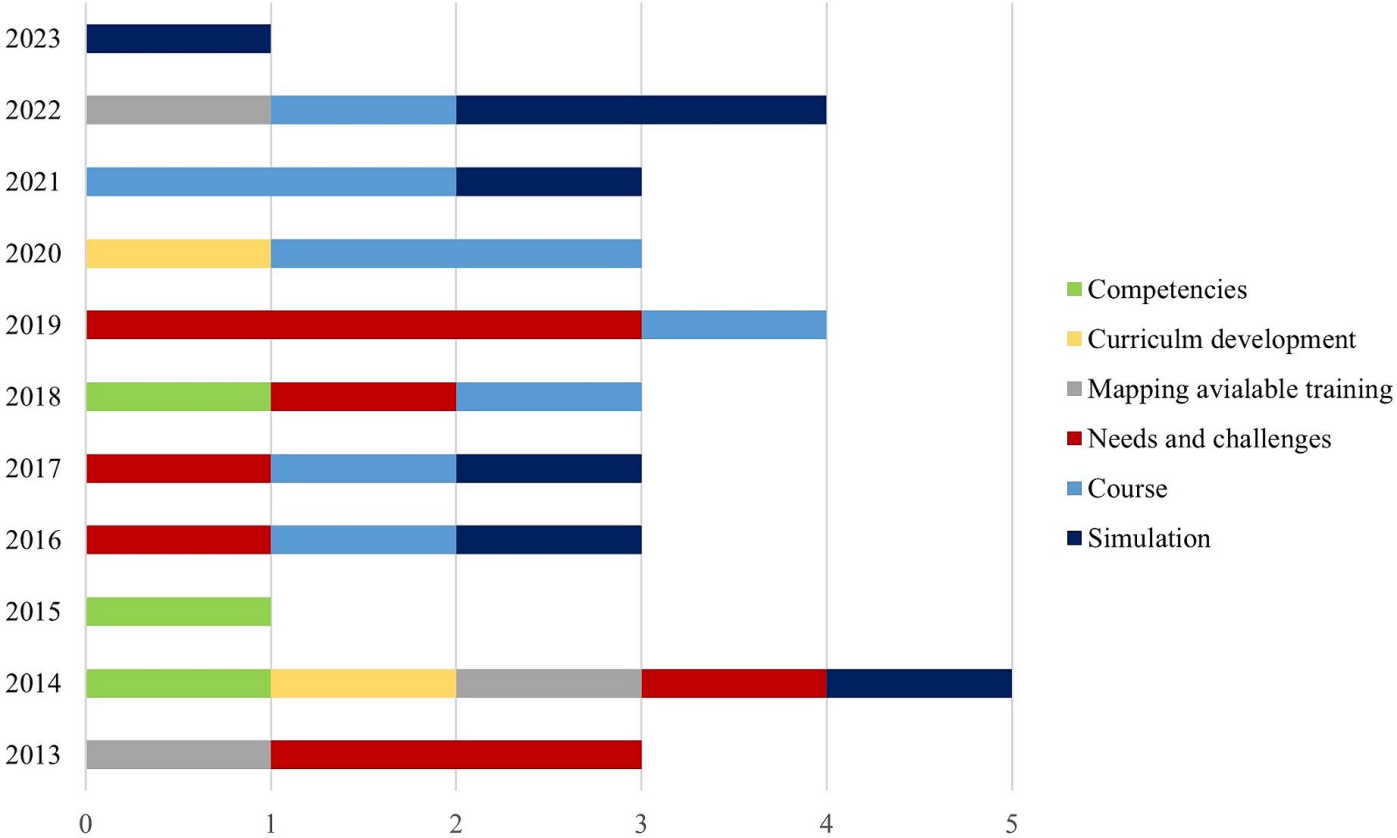
Trends of humanitarian health training publication.

On average, three publications were published per year concerning humanitarian health education. Most articles focused on the needs and challenges of humanitarian health education, which were the dominant themes between 2013 and 2019, followed by the description and/or evaluation of courses or simulation articles that dominated publications starting from 2016 (Figure 2).

About 25% of the studies were expert opinions (19, 20, 31–34) or narrative descriptions without clarifying the research methods that were used (35–37). Reviews were mainly applied to map the existing training opportunities (27, 38), competencies (29), and qualification guidelines (39). With interviews, literature reviews were also used to develop training frameworks (40), curricula (41), and competencies (42, 43). The mixed-methods study design was used mainly to evaluate the training (28, 44–51). The study design of the included studies is described in Table 2. More details concerning the characteristics of the included studies can be found in Supplementary material 2.

**Table 2:** Type of the study.

Mixed (quantitative and qualitative) (28, 44–51) Cross-sectional survey (52) Commentary, Editorial, Perspectives, Viewpoint (19, 20, 31–33, 35, 36), Single Interview (34), Short report (53), Narrative description (54) Literature search (38–43) Interviews (40–43, 53, 55) Systematic review (29, 39) Web-based review (27, 38) Analysis (though the methods was not clearly specified) (37) Observations (56)

The themes of the included studies revolved around training needs (19, 20, 31–33, 37, 39, 40, 55) framework (29, 39–43, 52) education and training mapping (12, 27, 38) and humanitarian health education and training programs, which include courses description (35, 36, 44, 45, 49, 54) and evaluation (45, 48, 49), simulation to train participants (46, 50, 51, 56), or to assess their competencies pre-deployment (28, 34, 47).

### 3.2 Education and training needs and challenges

The need for humanitarian health professionalization for global health security was emphasized (19). The movement of humanitarian health professionalization proposed certification for entry-, mid- and higher-level candidates through competency-based training and competency verification. The competencies needed included professional and technical competencies (12, 19, 31, 40), context adaptation (19, 40), core humanitarian competencies (12, 19, 31), and team performance (40). Competencies could be verified through examination, experience, and affiliation with professional associations (19).

However, no consensus-based humanitarian health competencies or curricula (27, 38) were found, nor guidelines defining the qualification of and preparations for international participants in sudden-onset disaster response in the health sector (39). The curriculum design for humanitarian health education and training was rarely reported. Identified curriculum design was either competency-based or was taught within a competency-based, subject-based, or outcome-based curriculum (27). The competencies used to develop the curriculum were discipline-specific but not humanitarian-specific (12). A systematic review identified several competencies related to disaster medicine or the humanitarian field – such as resources management, logistics, coordination, and childcare clinical skills – but no competency framework was found for humanitarian health (29).

Recommendations for training development included advanced training courses in operational public health (19), advanced clinical skills (20), advanced training in international humanitarian law and the Geneva Convention (20, 33), negotiation, violation reporting, and health services design and management (20). Needs assessment, nutrition and food security, safety and security, monitoring and evaluation, water supply and sanitation, refugees and human rights, protection, and logistics, were also identified as essential topics for humanitarian health training (12).

Reported challenges for professionalization included inaccessibility to education and training opportunities, especially for the local responders from the Global South due to the concentration of face-to-face training in the Global North (27), high training costs for low-income countries’ students (12, 27) and predominance of theoretical teaching methods and assessment of existing training (27).

To address existing training gaps, the literature has emphasized the need for experiential learning (27, 34, 55), distance learning – especially for mental health training (32), and combining both modalities using virtual simulation (27, 51). Literature also underscored the importance of evaluating the effectiveness and efficacy of existing courses and simulations through documentation collection, pre-deployment training, and after-action (37).

### 3.3 Mapping education and training opportunities

Burkle et al., Jacquet et al., and Bahattab et al., mapped and described the characteristics of humanitarian health education and training programs (12, 27, 38). Burkle et al. mapped training centers in North America (12), while Bahattab et al., and Jacquet et al., mapped humanitarian health education and training worldwide (27, 38). The number of identified training and education programs was 12 (12), 21 (38), and 146 (27), respectively.

Information reported in these publications include the training providers (12, 27, 38), year of establishment (12, 38), location (12, 27, 38), program funding (12) target audience (12, 27, 38), prerequisites (27), course composition (12), qualification (12, 27), curriculum design (12, 27, 38), content (12, 27), duration (12, 27, 38), delivery modality (12, 27), teaching and assessment methods (27), and tuition fees (12, 27, 38).

The identified humanitarian health education and training programs were varied when it comes to the target audience, content, and duration (12, 27, 38), with the majority of courses being short (27). Concerning the mode of delivery, courses were organized face-to-face, online (12, 27), or in a blended (27) format. Most education and training programs were based on theoretical teaching and assessment (27).

### 3.4 Curriculum

The curriculum development process was described only in two articles (41, 52), and both reported curriculum development for bioethics courses (41, 52). While the first article used a survey to identify the needs of medical students (52), the second one conducted a systematic review to guide the development of the curriculum, which has been implemented pre-mission and evaluated by comparing the trainees’ essays post-mission with the core competencies of the Accreditation Council for Graduate Medical Education (ACGME) (41).

The Sphere Handbook guided curriculum development for courses (44, 53), and simulations (50). The simulation curriculum also covered Core Humanitarian Standards, humanitarian and human rights law, rapid assessment, security, Geographic Information Systems, leadership, disaster medicine, and psychological first aid (50). The materials from “Health Emergencies in Large Populations” were used to develop a curriculum for humanitarian health courses (12).

### 3.5 Competency framework and skills

Competency-framework development was the main focus of only two articles, which created core competencies for nutritionists (43) and technical competencies for pharmacists (42). The development of the framework relied on literature review and expert interviews (42, 43).

Competencies for specific courses were developed using relevant literature on international education frameworks for disaster and public health emergencies (45, 49) such as the Sphere Handbook and the International Council of Nurses Framework for Disaster Nursing (54). Dickey et al. did not explicitly specify the competencies used to guide the course development but identified the participants’ post-training self-reported competencies (36). Learning objectives linked to The Core Humanitarian Competencies Framework were used to develop tools to evaluate participants’ performance during simulation (28, 47), and also employed for simulation development (28, 46, 47, 50, 51, 56) without references to competency framework.

### 3.6 Training programs: courses and simulations

Depending on the target audience and duration, different humanitarian public health topics were addressed by the training programs described in the retrieved articles. Communication for epidemic (36) was the sole focus of one course (36). Psychological support and various communication-related topics were covered by other trainings (12, 35, 44, 45, 49). Another course focused on civil-military interoperability during complex humanitarian emergencies (53). In other cases, the course content included the Sphere Handbook’s humanitarian standards (44, 45, 54).

Among the articles addressing simulation, team training was the focus of military teams (56) and international Emergency Medical Teams (EMTs) (46). Though different simulation scenarios were reported, all of them involved humanitarian settings such as a tsunami (28), a major earthquake in a low-income country (46), complex emergencies (51), conflict-based response for civilians (50), or military battlefield (56). According to the aim and scope of the simulation, participants were expected to perform tasks such as need assessment (28, 46, 50, 51) or propose a response plan (50, 51). Moreover, the humanitarian competencies of the participants were evaluated by asking them to perform tasks such as distributing food (28), managing water and sanitation (28), attending UN meetings, following security commands, organizing vaccination campaigns, and evacuating (28, 34), or react to a situation such as a roadblock, or ambush (28).

#### 3.6.1 Target audience for the identified courses

Among the training identified, only one targeted undergraduate students (54), while the rest targeted graduate students and health professionals (36, 44, 45, 48, 49, 54), such as nursing students (54), senior residents (49), public health graduate students (35, 36), medical and health professionals (36, 44, 45), or international civilian and military personnel (53).

#### 3.6.2 Target audience for the identified simulation

In the case of simulations, the targeted audience included military undergraduate medical, nursing, and psychology students (56), graduate students from different backgrounds (28, 50, 51), health professionals (34), humanitarian professionals (47), and EMTs (physicians, nurses, logisticians, coordinators, etc.) (46).

#### 3.6.3 Teaching delivery modality

Blended methods were the most common delivery modality for the humanitarian health courses included in this review (36, 44, 49, 53, 54). One of the courses was delivered entirely online (48), the other two were conducted face-to-face (45, 53), while in one course, the delivery modality was unclear (35).

Simulations were conducted face-to-face (28, 34, 46, 47, 50, 56) except for one (51), which was delivered virtually by adapting the conventional face-to-face modality. The pre-simulation training and teaching material were delivered to the trainees either online or through blended methods (46, 50).

#### 3.6.4 Teaching strategy and methods

The courses used multiple teaching methods and strategies, including frontal lectures, (36, 44, 45, 54) video-lectures, (49, 54) or lectures based on pdf material, and interactive sessions such as case studies (54), group work and discussions (36, 44, 45, 49, 54), presentations (54), assignments (36) and formative quizzes (48). The course described by Quinn et al. relied on a collaborative problem based on a learning approach that uses discussion and sharing experience (53).

Simulations were also used as teaching methods. The simulation types used for teaching were table-top exercises (34, 36, 44–46, 49, 54), multiplayer virtual simulation (49), full-scale (56), field-based (34), and operational functional exercises (46). Field-based simulations, on the other hand, were used as evaluation methods (28, 47). Further information about courses and simulation can be found in Supplementary material 3.

#### 3.6.5 Student assessment

Methods to assess trainees included attendance (44), post-course knowledge (45, 48, 49), and/or behavior assessment by the field supervisor (49).

Facilitators assessed the participants’ performance during the simulation to determine their readiness for deployment (47). An electronic tool was used to compare the competencies scores and global rating scores between evaluator assessments, peer evaluations, and self-evaluations (28).

#### 3.6.6 Training evaluation

The Kirkpatrick evaluation framework was used to evaluate the effectiveness of the training (49), while Greenhalgh et al.’s quality framework and the Donabedian model were employed to evaluate the quality of the training (48).

The methods used to evaluate the training courses were feedback (36, 44, 53) or satisfaction survey (45, 49) change in pre-post-test score of objective knowledge (45, 49), and follow-up of students behavior reported by the students themselves (45) or their supervisor (49). Other methods for course evaluation were evaluating the structure and format of web-based course, students data such as assessment scores (quiz results), incoming student survey and outgoing student survey, dropout student survey, staff data, semi-structured staff interview (with tutors and with course directors), staff curricula vitae (48) and facilitator roundtable (53).

Simulation training effectiveness was evaluated by assessing different outcomes, including trainees’ performance as individuals (50) or as a team (46), and conversion of field simulation into a virtual setting (51), or translation of interprofessional military knowledge into civilian education (56). Different evaluation methods – depending to the aim of the simulation aim – were used, including observation (56), pre- and post-simulation tests (50), learning self-assessments (50), before and after individual members’ perceptions of teams’ self-efficacy, teamwork skills (46), trainees feedback (34, 47, 50, 51) evaluator feedback (28), follow-up interview (47), and trainees versus trainers quality of training (46).

### 3.7 Success and challenges of the identified training programs

#### 3.7.1 Successful characteristics of the identified programs

The identified training programs reported characteristics and strategies that can improve training outcomes, cost-effectiveness, accountability, quality, flexibility, adaptability, global participation, and participant engagement.

##### 3.7.1.1 Collaborative partnership and interdisciplinary participation

Several courses reported collaborative course development involving collaboration with various stakeholders, including academic institutions and various entities such as other academic institution (35), governmental (35, 45, 54), private educational organizations (35), UN organizations (36, 47), humanitarian organizations (35, 49), civil-military organization (53), and local host country governments (36), and academic institutes (44). This partnerships can exchange expertise and bridge the gaps between the academia and field operation (49, 53), allowing for efficient use of resources (44), promoting interoperability between civil- and military respondents (53) and providing networking opportunities for students (35).

Most programs target participants from diverse professional disciplines and expertise (28, 36, 45, 46, 48, 50, 53). This approach fosters collaboration, accountability, and communication skills, facilitates understanding of broader perspectives, and enables multi-directional learning (36). Some of the courses adapted their training to accommodate participants at different levels (35, 45, 48), or envision to do so (53).

##### 3.7.1.2 Mixed teaching methods and experiential learning

Cognitive engagement was enhanced through a combination of different pedagogical approaches, including theoretical background and experiential learning through either field experience (35, 49) or simulation, which was a prominent feature of the identified training courses (36, 44, 45, 47, 49, 53).

Realistic scenarios and simulation exercises are effective methods for teaching and evaluating performance and operational skills that cannot be taught using other methods (28, 34, 36, 44–47, 49, 54, 56).

Simulation was also used for interprofessional team training by creating a supportive learning environment, promoting teamwork, and fostering respect for diverse roles within interprofessional teams (56). Effective simulation design strategies include balancing realistic, high-fidelity simulations (50) with practical field experience, ensuring trainees’ safety (35), avoiding distressing experiences, and providing mental health care during simulations (34). Other effective strategies include iterative design process, simulation implementation management, and providing immediate feedback and debriefing about participants’ performance (50).

##### 3.7.1.3 Mixed modality and technology adaption

Most identified training programs used a mix of online and in-person teaching modalities (36, 44, 46, 49, 50, 53, 54). The use of flipped-class room promoted the flexibility, engagement and interactivity between the faculty and participants (48, 49, 54). Online training is a cost-effective and sustainable modality that allows for global participation (46, 48, 51, 54), and can be used either as a standalone method or to prepare participants for in-person training (36) allowing for more time for interactive learning (53). Online modalities can be also used to deliver simulations (51). Technology can also be efficiently used for student assessment using either online (48) or offline methods (28).

##### 3.7.1.4 Evaluation

Training program evaluation and participant assessment, using different scope of evaluation, were employed to assess and validate training quality (46, 48), effectiveness of outcomes (28, 34, 36, 44–51, 53), and impact (36).

#### 3.7.2 Challenges, gaps, and lessons learned

The identified humanitarian training programs encountered several gaps and constraints, which can be categorized as logistical and technical. These challenges may impact the implementation, sustainability, quality, and accountability of these programs.

The lack of recognized standards for curriculum or competencies limits the development and evaluation of humanitarian health trainings or the assessment of trainees’ performance (28, 49). Most training programs evaluations were limited due to the lack of evaluation frameworks (48). The evaluation results were limited due to several factors: the assessment focused on short-term outcomes (47, 49), the subjective nature of the evaluation (47), limited follow-up feedback (47), and limited generalizability due to the small sample of the participants (49, 56), the scope of evaluation (49, 56) or the modality of evaluation (51). To ensure accountability in the field, there is the need to develop standardized and validated competency and training evaluation frameworks (47).

The development, implementation, evaluation, and performance assessment of training programs, especially field simulations, are resource-consuming and associated with high time, financial and logistical burdens, often resulting in short duration for these training programs (44, 45, 49, 51). Localization of training (44) and the use of online modalities to share and deliver training, including simulations, are sustainable and cost-effective methods that enable global participation (46, 48, 51).

Technological limitations associated with online modalities include poor internet connections (44, 45), and challenges in communication due to time zone differences (51), which were barriers to engagement. To overcome these barriers, some programs used offline, downloadable materials, or low-bandwidth materials to balance interactivity and accessibility (48). Another limitation associated with technology use is that e-learning may not always be suitable for transferring practical skills (48). Nonetheless, e-simulation can address this limitation (51).

Finally, the English language posed a barrier for local responders from Haiti to attend the course, prompting recommendations to conduct future courses entirely in local languages (36).

## 4 Discussion

This scoping review provided an overview of the state-of-the-art of peer-reviewed literature on humanitarian health education and training published during the last decade. The results summarized the peer-reviewed publication characteristics and their content concerning humanitarian health training and education. The findings of this study could serve as a starting point for the development of further training opportunities and to address the identified gaps in research.

The study identified 32 articles that focused on training needs (19, 20, 32, 33, 37, 39, 40, 55) and frameworks (29, 41–43, 52), mapping of education and training opportunities (20, 27, 38), competencies evaluation, and courses or simulations descriptions and/or evaluation (28, 34–36, 44–51, 53, 54, 56).

Although the articles retrieved showed significant variation in focus, study design, and outcomes measured, it is possible to identify some trends. For example, most articles focused on the needs and gaps, while more recent publications focused on the description and evaluation of training courses and simulations. Moreover, articles have shown a growing number of opportunities for humanitarian health education and training over the last decade at the global level (27, 38). This trend may have a positive effect on trainees, donors, and aid recipients. Still, the inequity of program proliferation in the Global North perpetuate an imbalance of power (27), as the hiring process may advantage international staff, who have physical and financial access to the training and educational programs, over the national staff. Training of national health staff is essential to localize humanitarian aid and to leverage equal opportunities for humanitarian health professionals worldwide. This task could be achieved through e-learning development (27), sharing training curriculum and materials through online platforms (46, 57), developing regional training initiatives (57), and capacity-building of national academic institutions through North–South partnerships as well as academic-non-governmental organizations partnerships (58). All these methods are not only effective, but also efficient (44, 48) and sustainable (46, 59) These partnerships must be driven by local leaderships to ensure the contextualization and sensitivity to local responders (57, 59, 60). Furthermore, initiatives promoting open e-simulation are emerging, which can further support these efforts by providing practical, accessible, and cost-effective training solutions (61, 62).

The findings of this review showed growing trends in the use of simulation in humanitarian health courses (36, 44, 45, 49, 54), and an increase in the number of articles that describe and evaluate simulations (46, 56) or evaluating competencies using simulation (28, 47). However, the increased number of publications focusing on simulation does not necessarily reflect the general current practices, especially for e-simulation, where its use in training and evaluation for humanitarian health remains limited (27). Nevertheless, the trend of publishing innovative practices reflects the future directions and will enable educators to replicate and adapt such practices worldwide, as well as the transfer of knowledge.

Despite the emphasis on the need for competency-based training (19, 20, 40), there is still no consensus for standard competency or curriculum (27, 38). This is also reflected by the absence of qualification guidelines specifying the necessary competencies for humanitarian health workers (39), and the lack of any agreed-upon accountability mechanisms for verifying these competencies. While most organizations have their own competency framework, and some have competencies related to humanitarian health (63–65), or even mention that these frameworks can guide training (66, 67), the evidence is limited on how these frameworks have been applied to guide the development and evaluation of training programs. Furthermore, when these competencies were used to develop training courses, they were adapted from existing frameworks related to disaster medicine and public health (45, 49). Alternatively, training objectives were used to measure the training outcomes in simulation without reporting competency framework from which these objectives were derived.

The lack of a standardized curriculum and competency framework will remain an issue for the credibility and quality of humanitarian training. A recent survey has shown that the majority of recruiters in humanitarian organizations would favor experience over qualification (68), a practice that raises questions about humanitarian respondents’ accountability, but it may also reflect the lack of trust in the academic sector to satisfy the evolving needs in the field. The findings revealed that the core humanitarian competency framework (69), which is not specific to health, was endorsed and used to evaluate the humanitarian competencies during humanitarian health training (28, 47), which reveal that there is a recognized needs for core humanitarian competencies apart from technical skills. Moreover, the Sphere Handbook (70), which is recognized for its common principles and universal minimum standards for humanitarian response, was frequently used by different training, either for curriculum or competency development or for teaching specific topics (44, 45, 50, 54). This reflects that these documents and their frameworks are recognized well among academic as well as humanitarian and can serve as a starting point to develop consensus among different training stakeholders.

Furthermore, few training reported assessment and evaluation framework, an important tool for quality and accountability (48, 49). The lack of evaluation frameworks, especially for e-learning (28, 49, 71) and challenges in assessing training outcomes were considered barriers to conducting such evaluations (60). Future research should focus on developing standardized metrics for evaluating the effectiveness of humanitarian health education and training. Additionally, more research is needed to understand how these programs can be scaled up and sustained over time.

**Figure 3:**
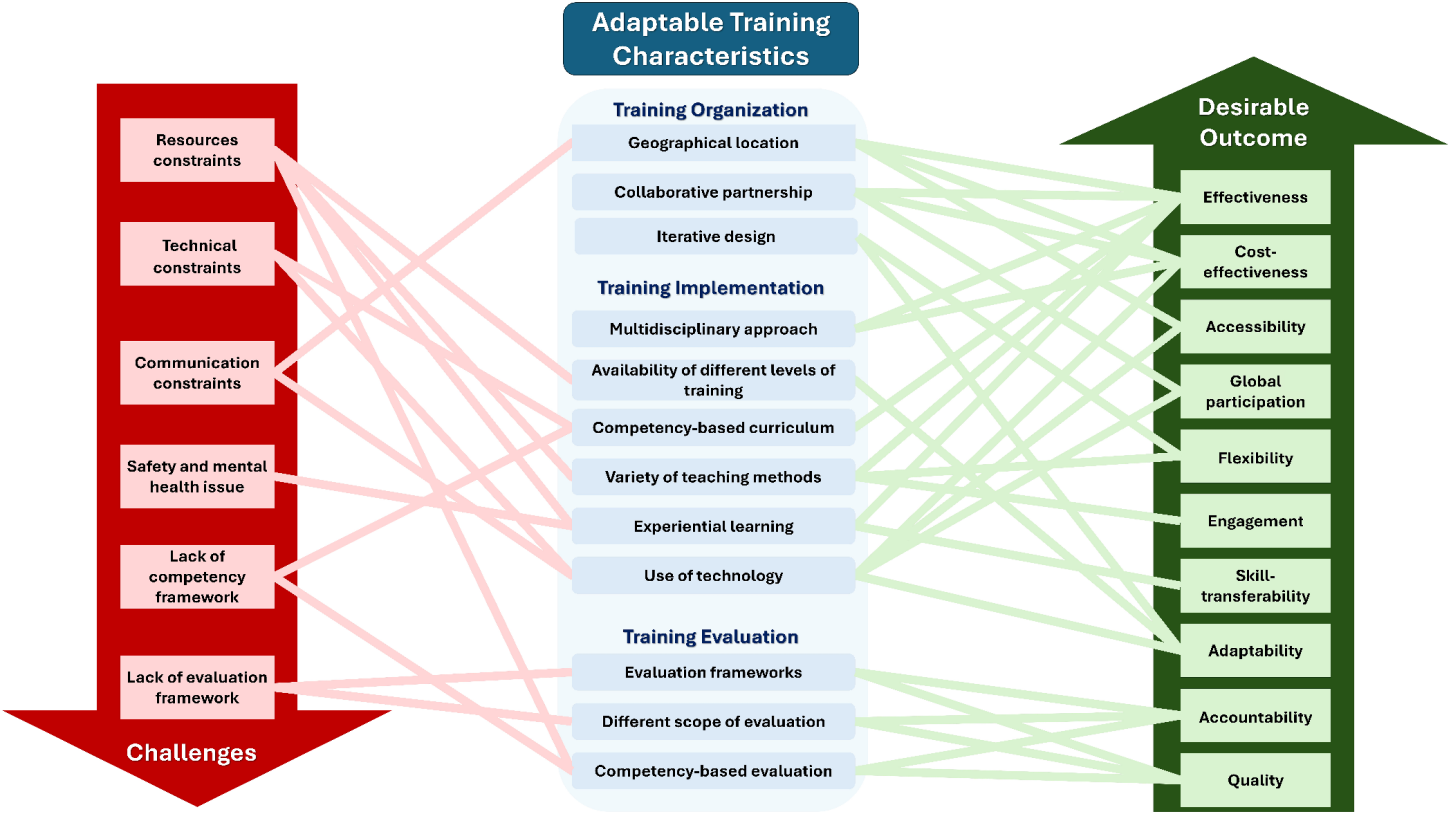
Adaptable training characteristics, their desirable outcomes, and challenges for their implementation.

Beside the gaps and challenges, the identified training programs have shown several characteristics that can be adapted by other humanitarian health training initiatives to improve training accessibility, effectiveness, cost-effectiveness, flexibility, adaptability, skill transferability, engagement, and accountability (Figure 3). Collaborative, interdisciplinary, experiential learning were prominent features contributing to the success of training programs. A collaborative partnership between academic, governmental, and other sectors, including the military, offers several benefits and can help overcome the barriers associated with many training challenges. These advantages include the efficient use of resources and expertise, bidirectional and peer learning, the provision of realistic humanitarian settings for interaction when combined effectively with proper teaching methods such as simulation, and networking and growing opportunities that can have a significant impact on responses in the field. However, without localization of humanitarian training initiatives, through locally driven leadership and partnership, the impact of these training initiative will likely fail to address specific local needs and contexts, and will perpetuate a neo-colonialist approach, making the interventions less relevant and less beneficial to the humanitarian respondents undermining their effectiveness and sustainability.

### 4.1 Limitations

This study has some limitations. First, this review was limited to English-language peer-reviewed articles, any relevant article written in other languages were not included. Second, since gray literature is heterogeneous, it mainly overlaps with other fields, has more diverse purposes, and – compared to peer-reviewed literature – it usually focuses on a single organization’s performance and its capacity building, it was not included in this review that aimed to provide a more generalized overview. In addition, this review does not aim to map the existing competencies, curriculum, or courses. Rather, this review aims to provide a generalized overview of the current thinking on humanitarian health education and training, which can stimulate future research and training development, and followed the recommended standards for the systematic methodology for conducting scoping reviews, which ensures transparency and reproducibility.

## 5 Conclusion

This review summarized the current state-of-the-art peer-reviewed literature on humanitarian health education and training. It identified trends over the last decade and key areas for future educational research and development. Despite the increase in training opportunities, several gaps, and opportunities to improve the quality and learning experience of humanitarian health education and training were identified. Simulation is still limited as a teaching method. Recent trends in reporting courses and simulations can facilitate lessons learned and best practices for training programs development and evaluation. However, standardized competency and curriculum frameworks are needed to ensure the quality and credibility of humanitarian health professionals. Evaluation of training conducted by current programs is also limited within the existing literature. Evaluation using a standardized framework and metrics would contribute to improving the quality and long-term sustainability of education and training programs and constitutes an important area for future research. The findings of this review support interdisciplinary, collaborative partnerships to address these gaps and develop future training.

Furthermore, training initiatives should prioritize local staff training support, through fostering regional centers and local institutions’ leadership and investing in accessible e-learning, including e-simulation. The review also highlighted the need for continued research, reporting innovation, and evaluation of humanitarian health education and training.

## Data availability statement

The original contributions presented in the study are included in the article/Supplementary material, further inquiries can be directed to the corresponding author.

## Author contributions

AB: Conceptualization, Data curation, Formal analysis, Investigation, Methodology, Writing – original draft, Writing – review & editing. MT: Data curation, Investigation, Writing – review & editing. IH: Supervision, Writing – review & editing. FD: Resources, Supervision, Writing – review & editing. LR: Resources, Supervision, Writing – review & editing.

## Supplementary material

SUPPLEMENTARY MATERIAL 1 Database search terms and search queries.

SUPPLEMENTARY MATERIAL 2 Characteristics and aim of the selected articles.

SUPPLEMENTARY MATERIAL 3 Characteristics of the identified courses and simulation.
