# Supplementary material for: Humanitarian health education and training state-of-the-art: a scoping review
Source: Front Public Health. 2024 Jul 29;12:1343867. doi: 10.3389/fpubh.2024.1343867 (PMC11317244; doi:10.3389/fpubh.2024.1343867)
Supplement: SUPPLEMENTARY MATERIAL 1 — Database search terms and search queries. [file Table_1.DOCX]

**Additional file 1:**

**Search terms and search queries**

| **Search terms and search queries for PubMed**  PubMed via the National Library of Medicine  **Search run 14/02/2023, updated 27/03/2023** | | |
| --- | --- | --- |
| **Search term** | **Query** | **Item found** |
| #1 Humanitarian health | (((((humanitarian public health) NOT (clinical)) OR (humanitarian health)) AND (((international humanitarian response OR armed conflict*[tiab] OR combat zone*[tiab] OR conflict affected[tiab] OR conflict related[tiab] OR conflict setting*[tiab] OR conflict zone*[tiab] OR humanitarian[tiab] OR military conflict*[tiab] OR military medicine[tiab] OR relief work[tiab] OR war[tiab] OR warfare[tiab] OR wars[tiab] OR wartime[tiab] OR warzone[tiab] OR doctors without borders[tw] OR medecins sans frontieres[tw] OR red cross[tw] OR "Warfare and Armed Conflicts"[Mesh:NoExp] OR "Armed Conflicts"[Mesh:NoExp] OR "Warfare"[Mesh] OR "Military Medicine"[Mesh] OR "Military Personnel"[Mesh] OR "Medical Missions"[Mesh] OR "War-Related Injuries"[Mesh] OR "Relief Work"[Mesh] OR "Hospitals, Military"[Mesh] OR "War Exposure"[Mesh]))) | 5170 |
| #2 Education/Training | (((competenc*[tiab] OR curricula*[tiab] OR curriculum*[tiab] OR educate*[tiab] OR education*[tw] OR medical instruction[tiab] OR medical interns*[tiab] OR medical residen*[tiab] OR preparedness[tiab] OR residency[tiab] OR taught[tiab] OR teach*[tiab] OR train*[tiab] OR "Education"[Mesh:NoExp] OR "education" [Subheading] OR "Education, Medical"[Mesh:NoExp] OR "Education, Medical, Continuing"[Mesh] OR "Education, Medical, Graduate"[Mesh] OR "Curriculum"[Mesh] OR "Teaching"[Mesh] OR "Hospitals, Teaching"[Mesh:NoExp]))) | 1,733,657 |
| #3 Humanitarian health education | #1 AND #2 | 1383 |
| Filter | Filters: English, from 2013 - 2023 Sort by: Most Recent | 957 |
| **Search terms and search queries from other databases 27/03/2023** | | |
| **Scopus** | ( ( humanitarian  AND health )  AND  ( training  OR  education  W/  curriculum  OR  competency  OR  framework  OR  course ) )  AND  PUBYEAR  >  2012  AND  PUBYEAR  <  2024  AND  ( LIMIT-TO ( DOCTYPE ,  "ar" ) )  AND  ( LIMIT-TO ( SRCTYPE ,  "j" ) )  AND  ( LIMIT-TO ( LANGUAGE ,  "English" ) ) | 504 |
| **Web of Science** | humanitarian health (Topic) and education OR training (Topic) | 777 |
| **ERIC** | ( humanitarian health or humanitarian public health or relief work ) AND ( education or training ) | 48 |
