# Supplementary material for: Humanitarian health education and training state-of-the-art: a scoping review
Source: Front Public Health. 2024 Jul 29;12:1343867. doi: 10.3389/fpubh.2024.1343867 (PMC11317244; doi:10.3389/fpubh.2024.1343867)
Supplement: SUPPLEMENTARY MATERIAL 3 — Characteristics of the identified courses and simulation. [file Table_3.DOCX]

**Table: Characteristics of the identified courses**

| **no.** | **Author** | **Main topic** | **Training provider** | **Location** | **Duration** | **Targeted audience** |
| --- | --- | --- | --- | --- | --- | --- |
| 1 | (Dickey et al., 2021) | Behavioral Communication Strategies for Global Epidemics | New York University (USA)vand UNICEF | Lebanon  offered four other times— in New York City (2015 and 2016), Ghana (2017), and Nepal (2018). | 10 days | Participants from United Nations agencies, governments, and MPH (Master of Public Health) students from New York University’s School of Global Public Health (GPH).  *Mix of post-graduate students and professionals* |
| 2 | (Ripoll-gallardo et al., 2020) | Disaster medicine, public health, safety and security, infectious diseases, psychological support, communication, humanitarian law, leadership, and job-specific skills | CRIMEDIM  and MS, Italy | Italy | - 3-month distance learning module; - 1-week instructor-led coaching; - Field placement with MSF. | National and international senior residents (IV-V year) in anaesthesia & critical care, emergency medicine, and paediatrics.  *Postgraduate students (resident)* |
| 3 | (Bustamante et al., 2020) | Key components, core principles, and defined minimum standards of Sphere Handbook: | Brigham and Women’s Hospital (Boston, Massachusetts USA) collaborated with Hôpital Universitaire de Mirebalais’ and Department of Medical Education and Emergency Medicine (EM) residency program, Haiti. | Haiti | - Pre-course E-learning of 15 hours - 11.5 hours of lectures with simulation activities - 5.5 hours of interactive group exercises. | Haitian hospitals EM physicians and nurse prioritized  Other hospital services (medicine, paediatrics, surgery, orthopaedics, intensive care unit, rehabilitation, pharmacy, and mental health) were invited to send one to four participants, depending on their department’s size.  *Professionals* |
| 4 | (Bajow et al., 2019) | Public health, and different subject areas of humanitarian actions | The course was prepared by the Disaster Medicine Unit of the Mohammed Bin NaifMedical Center (Riyadh, Saudi Arabia) | KSA | 30 hours over a five-day period | Health care workers  *Professionals* |
| 5 | (Tam et al., 2018) | Public Health Principles | Hong Kong, \|China | Online | free 6-month program | Anyone with interest in disaster and medical humanitarian response, although it is aimed at  Postgraduate level. |
| 6 | (Williams & Downes, 2017) | Clinical care and Environmental, political, and sociocultural aspects of migration and conflict | United States; Emory and CDC, Italy | Atlanta, Georgia USA | Online modules and 3-day (total 24 hr). Two-credit course | Nursing students  Undergraduate Students |
| 7 | (D. P. Evans et al., 2016) | Certificate,  Field practicum: emergency response or preparedness  and  Fellowship | Center for Humanitarian Emergencies, Emory University | Atlanta, Georgia USA | Certificate: quasi-traditional, short-course format ranging from *two days to one week*  Field practicum: summer supervised practical experience  Fellowship over one year | Graduate and postgraduate students in Public Health  Certificate:  *Graduate Students*  Fellowship  *Postgraduate students (mid- career)*  *Professionals* |
| 8 | (Quinn et al., 2022) | International guidelines, concepts, and principles and their application to complex emergencies. Basic definitions focused on the complex relations and interdependence of health and human security. | The Kofi Annan International Peacekeeping Training Centre (KAIPTC) | Accra, Ghana. | One week (11-15 Nov 2019) | Senior Medical Officers and Force Medical Officers from the UN, the African Union, the Economic Community of West African States, The North Atlantic Treaty Organization, the European Union, NGOs; international civilian and military personnel preparing to serve on missions, crisis and disasters in support of best practices. |

**Table: Characteristics of the identified simulation**

| **No** | **Author** | **Type of simulation** | **Provider and location** | **Pre-simulation** | **Simulation scenario** | **Tasks** | **Duration** |
| --- | --- | --- | --- | --- | --- | --- | --- |
| 1 | (Kesler et al., 2022) | Full-scale simulation a large outdoor setting | University of Minnesota, United States | Pre-simulation online material and a brief series of lectures that covered basic concepts in humanitarianism | Conflict-based humanitarian disaster response | *conduct a rapid assessment.*  *present project proposals*, | 24 h |
| 2 | .(Kivlehan et al., 2021) | Virtual format using a combination of video conferencing, short messaging service, and cloud-based file storage software. | Harvard T. H. Chan School of Public Health, United States | The simulation exercise is the combined culmination of 2 courses: a 60-student graduate course on humanitarian response at the Harvard T. H. Chan School of Public Health and a 60-student professional course called the Humanitarian Response Intensive Course (HRIC). | Complex humanitarian emergency | conducting a *rapid assessment*  *preparing a service delivery plan* outlining their organization’s proposed response | 3-days |
| 3 | (Bodas et al., 2021) | Table-top and functional exercises | Europe -  (Turkey, Germany)  Package available free online. | The TEAMS Training Package is comprised of a set of 8 innovative blended-learning teaching materials and simulation-based exercises | Massive earthquake in LMIC | Standalone modules for scenarios throughout different phases of the humanitarian cycle (from pre-deployment till exist)   1. *Preparing for deployment* 2. *Arriving and Setting up* 3. *Setting priorities* 4. *Managing operational information* 5. *Responding to a mass casualty incident* 6. *Adapting practice to context* 7. *Planning the exit* 8. *Dealing with security threats* | 4- days |
| 4 | (Varpio et al., 2020) | Full-scale simulation  Military interprofessional education (MIPE) | at the reserve Army base located in Fort Indiantown Gap, Pennsylvania, United States | - | Battlefield deployment | *Variety of scenarios designed to test learners’ clinical and collaboration skills. Simulations included, for example:*  *1. convoying patients from point of injury to a forward aid station (i.e., a patient care facility operating out of a tent or temporary building),*  *2. caring for patients in forward aid stations, and*  *3. participating in a global health engagement activity with local villagers.* | 4-days (30.5 h) |
| 5 | .(Evans et al., 2017) | Humanitarian Competencies assessment during simulation | Camp Interval, Saint Lucie des Laurentides, Quebec, Canada | pre-requisite coursework prior to the SimEx. Coursework was done in one of three ways: (1) as part of Laval University’s (Quebec City, Quebec, Canada) Masters in Humanitarian Studies Program;26 (2) a 55-hour, in-class program at McGill University;27 or (3) a 2-week program that involved one week online28 and one week in-class. | Fictional *tsunami* | Participants worked in eight teams of 10 to:  conduct needs assessments, mapping,  communication, draft reports, give press conferences, attend cluster meetings, and work through different injects (skills stations) | 3-days (72-hours) |
| 6 | (Cranmer et al., 2014) | Humanitarian Competencies assessment during simulation | WHO collaborated with the Humanitarian Training Initiative (HTI) to create the Public Health Pre-Deploymen Course simulation exercise (SimEx)  Tunis, Tunisia | Two-week Public Health Pre-Deployment Course | - | - | 3-days |
| 7 | (Nouvet, 2016) | Full-scale simulation | Canadian Disaster and Humanitarian Response Training Program  Canada (location of the simulation not specified) | - | Complex humanitarian emergency | One night they do militia attack, which is quite scary. Sunday morning participants present their deliverables. They have to do media interviews, attend UN meetings, follow security commands, radio in, practice evacuating, organize a vaccination campaign. And all the while they’re being interrupted and they have to do all the skill stations and create situation reports. | 3-days (72-hours) |
